# Supplementary material for: Self-compassion and the impostor phenomenon: Associations and implications
Source: PLoS One. 2026 May 27;21(5):e0348905. doi: 10.1371/journal.pone.0348905 (PMC13215504; doi:10.1371/journal.pone.0348905)
Supplement: Attachment — Submitted filename: reviews_comments.docx [file pone.0348905.s002.docx]

Journal Requirements:

When submitting your revision, we need you to address these additional requirements.

1. Please ensure that your manuscript meets PLOS ONE's style requirements, including those for file naming. The PLOS ONE style templates can be found at

https://journals.plos.org/plosone/s/file?id=wjVg/PLOSOne_formatting_sample_main_body.pdf and

[https://journals.plos.org/plosone/s/file?id=ba62/PLOSOne_formatting_sample_title_authors_affiliations.pdf](https://mcas-proxyweb.mcas.ms/certificate-checker?login=false&originalUrl=https%3A%2F%2Ftrack.editorialmanager.com.mcas.ms%2FCL0%2Fhttps%3A%252F%252Fjournals.plos.org%252Fplosone%252Fs%252Ffile%253Fid%3Dba62%252FPLOSOne_formatting_sample_title_authors_affiliations.pdf%2F1%2F010f01988a993179-6ac41755-d2b7-4ee6-b319-d84490524b3c-000000%2FwqUeCwWiZbOXky29V5J2t94uGt-vlitlRWpsQTM9ZKU%3D223%3FMcasTsid%3D20893&McasCSRF=fe9253c5614238844ac83ef6beea786ede34dcfa23b956936b289d6a6519936b)

2. Please include a complete copy of PLOS’ questionnaire on inclusivity in global research in your revised manuscript. Our policy for research in this area aims to improve transparency in the reporting of research performed outside of researchers’ own country or community. The policy applies to researchers who have travelled to a different country to conduct research, research with Indigenous populations or their lands, and research on cultural artefacts. The questionnaire can also be requested at the journal’s discretion for any other submissions, even if these conditions are not met.  Please find more information on the policy and a link to download a blank copy of the questionnaire here: https://journals.plos.org/plosone/s/best-practices-in-research-reporting. Please upload a completed version of your questionnaire as Supporting Information when you resubmit your manuscript.

3. If the reviewer comments include a recommendation to cite specific previously published works, please review and evaluate these publications to determine whether they are relevant and should be cited. There is no requirement to cite these works unless the editor has indicated otherwise. 


Reviewers' comments:

Reviewer's Responses to Questions

**Comments to the Author**

1. Is the manuscript technically sound, and do the data support the conclusions?

The manuscript must describe a technically sound piece of scientific research with data that supports the conclusions. Experiments must have been conducted rigorously, with appropriate controls, replication, and sample sizes. The conclusions must be drawn appropriately based on the data presented.

Reviewer #1: Yes

Reviewer #2: Yes

Reviewer #3: No

2. Has the statistical analysis been performed appropriately and rigorously?

Reviewer #1: Yes

Reviewer #2: Yes

Reviewer #3: No

3. Have the authors made all data underlying the findings in their manuscript fully available?

The [PLOS Data policy](https://mcas-proxyweb.mcas.ms/certificate-checker?login=false&originalUrl=http%3A%2F%2Ftrack.editorialmanager.com.mcas.ms%2FCL0%2Fhttp%3A%252F%252Fwww.plosone.org%252Fstatic%252Fpolicies.action%2523sharing%2F1%2F010f01988a993179-6ac41755-d2b7-4ee6-b319-d84490524b3c-000000%2Fb14nIsxdKF0bT19HlWU1d-Wd5aJUZc3ca7AfUpYM7XA%3D223%3FMcasTsid%3D20893&McasCSRF=fe9253c5614238844ac83ef6beea786ede34dcfa23b956936b289d6a6519936b) requires authors to make all data underlying the findings described in their manuscript fully available without restriction, with rare exception (please refer to the Data Availability Statement in the manuscript PDF file). The data should be provided as part of the manuscript or its supporting information, or deposited to a public repository. For example, in addition to summary statistics, the data points behind means, medians and variance measures should be available. If there are restrictions on publicly sharing data—e.g. participant privacy or use of data from a third party—those must be specified.

Reviewer #1: Yes

Reviewer #2: Yes

Reviewer #3: No

4. Is the manuscript presented in an intelligible fashion and written in standard English?

PLOS ONE does not copyedit accepted manuscripts, so the language in submitted articles must be clear, correct, and unambiguous. Any typographical or grammatical errors should be corrected at revision, so please note any specific errors here.

Reviewer #1: No

Reviewer #2: Yes

Reviewer #3: No

5. Review Comments to the Author

Please use the space provided to explain your answers to the questions above. You may also include additional comments for the author, including concerns about dual publication, research ethics, or publication ethics. (Please upload your review as an attachment if it exceeds 20,000 characters)

Reviewer #1: Overall Summary:
This manuscript explores the relationship between self-compassion (SC) and the impostor phenomenon (IP) among Czech university students. The study uses the Impostor-Profile 30 (IPP30) and the Self-Compassion Scale (SCS) to analyze data from 601 students. The findings suggest that negative dimensions of self-compassion (like isolation and overidentification) are more strongly associated with IP, particularly with competence doubt.

Some Suggestions:
1. Abstract:
- The abstract provides a good overview, but could be more concise. Consider focusing on the most significant findings.
- Specify the type of statistical analysis used (e.g., correlational analysis, regression model, structural equation modelling).

***Authors’ response:***

*In accordance with the feedback, we revised the abstract to improve clarity and methodological transparency. We streamlined several sentences for clarity while retaining their original meaning; explicitly stated the types of analyses applied and clarified the SEM results.*

2. Introduction:
- The introduction effectively sets the stage by describing the impostor phenomenon and its relevance, especially in student populations.
- The rationale for focusing on university students could be further expand.

***Authors’ response:***

*We have expanded the rationale for focusing on university students by adding practical relevance and empirical support for our decision.*

3. Literature Review/Background:
- The literature review covers relevant concepts (impostor phenomenon, self-compassion) and instruments (IPP30, SCS).
- It could benefit from a more critical evaluation of existing literature, highlighting gaps the current study addresses.

***Authors’ response:***

*We have added a critical evaluation in the general description of the IP syndrome, highlighting the predominance of research on medical students. Further, in the section “IP and SC: well-being-related contexts and differences,” we emphasised the lack of studies examining the mutual relationships between these two concepts, which was already mentioned in the introduction.*

- In the section on Self-compassion, clarify how the IP and SC relate to reality.

***Authors’ response:***

*The description of relationships of the SC and IP to reality was added in the closing paragraph of the Self-compassion section.*

4. Methods:
- The description of participants, measures, and procedure is thorough.
- The sample size is justified, and the data collection method is appropriate.
- Mention the specific version of IBM SPSS Statistics used.

***Authors’ response:***

*The version of IBM SPSS Statistics was assigned more specifically.*

- Give reasons for using a double translation method from English to Czech. Expand on the forward-backward translation procedure.

***Authors’ response:***

*We are grateful to the reviewer for pointing out the improper description of the translation process. The information was corrected and specified.*

5. Results:
- The results section clearly presents the descriptive statistics and correlational analyses.
- The use of tables is effective for summarizing data.
- The SEM results should be reported with more detail (e.g., specific path coefficients, fit indices).

***Authors’ response:***

*We extended the interpretation of fit indices and added a specific description of the path coefficients.*

6. Discussion:
- The discussion relates the findings back to the hypotheses and existing literature.
- The discussion could be strengthened by addressing the limitations more explicitly and suggesting directions for future research.

***Authors’ response:***

*We have expanded the section on study limitations to include more specific details regarding the sample’s nationality, the reliance on self-report measures, and the cross-sectional design. Based on these additions, we also elaborated on potential directions for future research, highlighting the need for longitudinal and experimental approaches as well as alternative assessment methods (e.g., behavioral measures, informant reports). To strengthen this section, we incorporated recent scientific articles that further support* our discussion of limitations and methodological alternatives.

- Expand on the practical implications of the findings for interventions targeting impostorism.

***Authors’ response:***

*We thank the reviewer for this important suggestion. The section on practical implications has been extended in light of our findings and their discussion. Specifically, we now elaborate on the advantages of implementing self-compassion–centered interventions in group settings, which may help reduce students’ feelings of isolation and overidentification. We also highlight that embedding such interventions directly in the university environment could facilitate the transfer of self-compassionate responses into students’ daily academic lives, which are often shaped by high achievement orientation. Furthermore, we discuss the potential positive impact on students’ academic engagement and performance, as well as the broader benefits of incorporating elements of self-compassion–centered and mindfulness-based interventions into the university curriculum, particularly within courses addressing self-management and well-being at work.*

7. Tables and Figures:
- Ensure all tables and figures are properly labeled and referenced in the text.
- Figure 1 (path analysis model) is mentioned but not fully discussed in terms of its implications.

***Authors’ response:***

*The description of the SEM model was extended in the Results section, and further information on the practical implications was added in the Theoretical and Practical Implications section.*

8. References:
- Update references in 2024/2025 and check it is correctly formatted.
- Update the studies’ information and ensure they align with the context they were cited in.

***Authors’ response:***

*The references were updated and corrected in formatting where necessary.*

Specific Suggestions:
- Focus on Key Findings: In the abstract and discussion, highlight the most important and novel findings of the study.
- Address Limitations: Acknowledge the limitations of the study, such as the specific population (Czech university students), the cross-sectional design, and the potential for self-report bias.
- Expand on Implications: Discuss the implications of the findings for designing interventions to reduce impostor feelings and promote self-compassion, particularly in university settings.
- Suggest sending the manuscript for English proofreading before acceptance.

***Authors’ response:***

*The responses to the specific suggestions were mostly already addressed in the previous text. Furthermore, we appreciate the recommendation to have the manuscript proofread in English, which we have now done using the Editage proofreading service.*

Reviewer #2: Thank you for the opportunity to review your manuscript. You provide a compelling case for investigating the IP and SC relationship. The following are some thoughts to help take your work to the next level.
- I noticed that "self-concept", "self-attitude" and "self-image' are used in the manuscript, and in some instances, are used interchangeably - are they referring to the same or different concept(s)? Naive readers may not be familiar with nuances

***Authors’ response:***

*We thank the reviewer for this important note, and we acknowledge that readers might be confused by this kind of inconsistency. Therefore, we have decided to replace both self-image and self-concept with self-attitude, a broader term encompassing cognitive, emotional, and motivational dimensions (as inherent in the concept of attitude itself).*

- p. 3 line 39 - Why employers suffering from the IP?
- p. 8 line 165 - what is "mutual" correlation?
- p. 8 line 167 - should it be "lower levels of the impostor phenomenon..."?

- p. 8 line 178 - please provide % in addition to n's

***Authors’ response:***

*We corrected/removed all mistakes and redundancies and added* % where required.

- p.9 lines 197-198 - how do the six subscales map onto the core elements provide on p. 4 lines 78-79? To me, they appear quite different, not well aligned.

***Authors’ response:***

*The subscales do not match the core elements of the IP. For better clarity, we described all scales in the Research background section.*

- p.10 lines 210-213 - the logic between the two sentences is contradictory. Why would one use separate subscale scores in the absence of evidence for clear factor structure as reported in the literature??

***Authors’ response:***

*The use of separate subscale scores of the Self-Compassion Scale (SCS) is in line with the recommendations of the scale’s author (Neff, 2003; Neff, 2016), who explicitly allows the reporting of both total and subscale scores. Moreover, numerous empirical studies have analysed the subscales individually to capture the multidimensional nature of self-compassion. While some studies support a higher-order factor structure, others have highlighted methodological concerns regarding the validity of the total score (e.g., López et al., 2015; Muris et al., 2022). These authors recommend reporting partial scores rather than relying solely on the total score. Nevertheless, we thank the reviewer for the insightful comments that inspired us to clarify this in the Measures section.*

- p. 15 line 293-297 - - what theory is it based on? or is it purely based on the initial correlational analyses? That is, why for example, in Figure 1, self-kindness feeds into common humanity, and isolation feeds into overidentification?? The reviewed literature does not offer any justification for the model tested.

***Authors’ response:***

*Yes, the model is partially based on correlation analyses but is also grounded in theoretical considerations. For instance, Dreisoerner et al. (2020) note that “self-kindness may be linked to common humanity and isolation” (p. 25). They describe a vicious circle of self-criticism leading to shame, reduced socially oriented behaviour, and ultimately social exclusion. Furthermore, the connection between Isolation and Over-identification in our model is supported by Cleare et al. (2018), who reported the highest intercorrelation between these two subscales within the six-factor correlated model of the SCS. In addition to this theoretical foundation, our final model was selected after testing alternative models with poorer fit. We have now clarified this by adding information about the relevant literature both in the section on theoretical background and in the analytic plan.*

- p. 16 line 312 - should it be "....with scores in NEGATIVE subscales slightly prevailing"?

***Authors’ response:***

*We thank the reviewer for the important recommendation. This information is correct, but there was a typo in the Result section; we corrected it to ensure clarity and consistency.*

- p. 18 line 347 - what does "treatable" refer to??

***Authors’ response:***

*It was connected to the self-compassion level; we reformulated the text and replaced the improper verb with a better synonym.*

Reviewer #3: The present manuscript examines the relations between the Impostor Phenomenon and self-compassion in a sample of 601 students. The findings are mostly in line with expectations.

Overall, I was looking forward to reading the manuscript as the research question is interesting. However, my enthusiasm was dampened upon reading the manuscript. I provide several comments below. Taking my observations into account, the manuscript does not meet the standards for a contribution to the knowledge of the field. Accordingly, I recommend rejecting the manuscript for publication.

(1) Discrepancy between data and language: I am surprised that authors often provide causal interpretations and, thus, over-interpret the data and findings heavily. Considering the cross-sectional nature of the data assumptions about SC being a "protective factor" "influences etc. are impossible and inappropriate.

**Authors’ response:**

*We thank the reviewer for this valuable comment. We would like to clarify that our study never intended to make causal claims. Our theoretical framework and analytic approach were designed to examine associations, not causal mechanisms.*

*We acknowledge, however, that some of our original wording could have unintentionally suggested causal relationships. In response to the reviewer’s concern, we systematically revised the manuscript — including the title, abstract, introduction, results, discussion, and implications sections — to ensure that the language consistently reflects correlational, non-causal interpretations.*

*While the scientific claims of our study remain unchanged, we believe the revised wording now removes any risk of causal overinterpretation.*

(2) Introduction and literature review: Overall, the introduction reads very awkwardly and lacks flow that provides readers with a clear rationale of the study and the hypotheses. There are many instances where one-sentence paragraphs are given that appear to be disconnected from the remainder of the manuscript. Also there are several typos and grammatical errors that sometimes even alter the meaning of the words (e.g., "employers" vs "employees").

**Authors’ response:**

*We thank the reviewer for the feedback, which helped us identify and correct typographical and grammatical errors. We have also revised the structure of the Introduction and Research Background sections to improve coherence and provide a clearer rationale for the study. However, please note that the track-changes mode did not allow us to fully display certain revisions, especially those involving the restructuring and merging of paragraphs. These changes have nevertheless been carefully implemented in the manuscript.*
(3) Description of results: The results are displayed and reported very in transparently. For example, the tables contain all coefficients (e.g., one- and two-tailed tests of significance) and it is up to readers to find and seek the relevant coefficients. Also, there are many inconsistencies that are, again, up to the reader to solve (e.g., statistically significant with negligible effect size). The use of K-S tests for testing is questionable when considering the large sample size and how easily minor deviations from the normal distribution are detected. F-test is reported without degrees of freedom. RMSEA = .41 indicates are very ill-fitting model.

***Authors’ response:***

*Our original intention was to provide full transparency by reporting all coefficients from the statistical output. However, we acknowledge that this approach may have appeared overloaded and less reader-friendly. In response, we undertook a systematic revision of our results reporting to make it clearer and more consistent with APA guidelines. Further, we replaced the Kolmogorov–Smirnov test with the Shapiro–Wilk test, which is generally preferred for testing normality, and clarified in the manuscript that minor deviations were expected given the large sample size, but do not compromise the robustness of parametric analyses. We also added the degree of freedom in the F-test interpretation and included also the confidence intervals to be more consistent with recommendations of APA (7th edition).*

*We sincerely thank the reviewer for drawing attention to the value of RMSEA, which was mistakenly reported as 0.41 in the text due to a typographical. We would like to clarify that the correct value is* ***RMSEA = 0.041****, as was accurately presented in the table 7. We have revised the text accordingly to ensure correctness and consistency.*

(4) Mediation on basis of cross-sectional data: There is robust evidence that testing indirect effects/mediation analyses on basis of cross-sectional data produce biased parameter estimates (see the works by Maxwell and Cole; e.g., 2007). Hence, findings from this line of research can and should not be trusted.

Maxwell, S. E., & Cole, D. A. (2007). Bias in cross-sectional analyses of longitudinal mediation. Psychological methods, 12(1), 23–44. https://doi.org/10.1037/1082-989X.12.1.23

***Authors’ response:***

*We sincerely thank the reviewer for this important note. We fully agree that mediation analyses assume temporal ordering, which cannot be established with cross-sectional data. In line with Maxwell et al. (2011), we acknowledge that parameter estimates of indirect effects in such designs may be biased.*

*To address this, we carefully reviewed the entire manuscript (title, abstract, methods, results, discussion) and corrected any wording that might have implied causality. Terms such as “predictor,” “mediator,” or “influences” were replaced with non-causal alternatives (e.g., “associated with,” “indirect statistical pathway,” “correlate”). Further, we emphasised the exploratory, associative character of the relationships in the SEM model, which cannot be treated as evidence of causal mediation.*

*In the discussion, we explicitly acknowledge the limitations of cross-sectional mediation analysis and highlight the need for longitudinal or experimental research to validate the relationships between self-compassion and impostor phenomenon.*

*We hope these changes make clear that our study does not claim causal mediation, but rather provides associational findings consistent with theoretical models, to be tested in more rigorous longitudinal designs in future work.*

(5) Novelty: Even when taking the issues noted above aside, it is unclear what the novel contribution of this study is to the field.

***Authors’ response:***

*We have clarified the contribution of our study in the revised manuscript. Specifically, our work extends existing research in several ways:*

*It is based on a large and culturally underrepresented sample of Czech university students (N = 601), thereby broadening the evidence base beyond predominantly US/Western European contexts.*

*Unlike most prior studies that used the unidimensional Clance Impostor Phenomenon Scale, we employed the multidimensional Impostor-Profile 30. This allowed us to examine associations between specific IP dimensions and self-compassion (SC) subscales, rather than relying on a single global score.*

*Our findings show that negative SC dimensions (Isolation, Overidentification) are more strongly related to impostor feelings than positive SC dimensions (e.g., Self-kindness, Common Humanity). This nuance helps refine the theoretical understanding of how SC is linked to impostorism.*

*Finally, by identifying which SC subscales are most closely associated with IP, our study provides information that may be useful when considering the design or interpretation of SC-focused approaches in a student population vulnerable to stress and low self-efficacy.*

We hope that these revisions make the significance and novel contribution of our study clearer.

(6) While I applaud authors' efforts to provide a link to the open data, it does not work and leads nowhere.

***Authors’ response:***

*We are grateful to the reviewer for checking the connection with the Mendeley Data Repository, and we are sorry for the difficulties. To prevent such issues in the future, we replaced DOI with a direct link to the Mendeley Depository page.*
