# Supplementary material for: Self-compassion and the impostor phenomenon: Associations and implications
Source: PLoS One. 2026 May 27;21(5):e0348905. doi: 10.1371/journal.pone.0348905 (PMC13215504; doi:10.1371/journal.pone.0348905)
Supplement: Attachment — Submitted filename: reviews_comments2rev (1).docx [file pone.0348905.s003.docx]

**Response to Reviewers**

Reviewer #3: I thank the authors for addressing my comments in a careful revision of the manuscript. I have read the authors' responses and the manuscript to the comments closely and found that the manuscript was strengthened. However, some points require attention that should be addressed in a second revision of the manuscript:

(1) Authors note in the manuscript that the CIPS is unidimensional, which is not in line with the state-of-the-art of the literature, as the CIPS has been found to be presented by three dimensions in addition to a general factor (Brauer & Proyer, 2025). Hence, the claim on the "unidimensional scale method" when referring to the CIPS requires revision.

Brauer, K., & Proyer, R. T. (2025). Understanding the Clance Impostor Phenomenon Scale through the lens of a bifactor model. European Journal of Psychological Assessment, 41(2), 108–116. [https://doi.org/10.1027/1015-5759/a000786](https://mcas-proxyweb.mcas.ms/certificate-checker?login=false&originalUrl=https%3A%2F%2Fdoi.org.mcas.ms%2F10.1027%2F1015-5759%2Fa000786%3FMcasTsid%3D20893&McasCSRF=78d3209ff6942407aa27af2859fdac1485a4d3a9ab966573763b424d383a1865)

***Authors’ response:***

*We are grateful to the reviewer for drawing our attention to this recent and insightful study. Although we still have a rationale for using the IPP-30 instead of the CIPS in our research, we have gladly incorporated the cited paper into the manuscript to enhance the accuracy and clarity of our argumentation.*

(2) The IP is not a clinical construct. Hence, speaking of "symptomatology" is misleading and requires revision.

***Authors’ response:***

*We agree with the reviewer that the term “symptomatology” is somewhat inappropriate given the nature of the IP. In line with the study cited in the corresponding paragraph, we have replaced it with the term “syndrome.”*

(3) Tables should be formatted in line with APA recommendations instead of a copy-paste of the SPSS output.

***Authors’ response:***

*We are grateful to the reviewer for this helpful remark that has helped us to improve the graphical presentation and readability of the manuscript.
 In response, we have reformatted all tables in accordance with APA recommendations.*

(4) Asterisks *and* p-values in Table 5 are redundant. Please report either.

***Authors’ response:***

*Following the reviewer’s suggestion, we revised Table 5 (now Table 7) by removing the redundant “Sig.” rows and keeping only the significance asterisks, which aligns the table formatting with APA guidelines.*

(5) As authors note, the sample size is large, which relates to oversensitive tests of normal distribution (S-W test). Please report and interpret normality on basis of skewness and kurtosis parameters, as the S-W test is oversensitive to negligible deviations from the normal distribution. If those parameters are < 1, I suggest switching the correlation analyses to Pearson, as you do not analyze categorical data but continuous data in the sense of mean scores.

***Authors’ response:***

*We thank the reviewer for this valuable suggestion. In line with the recommendation, we assessed normality using skewness and kurtosis parameters, which indicated only minor deviations from normality. Given the large sample size and the approximately normal distribution of the data, we conducted the correlation analyses using Pearson’s correlation coefficient, which was considered appropriate for the continuous scores used in this study.*

(6) I have missed analyses regarding demographic variables such as age and gender.

***Authors’ response:***

*We thank the reviewer for this helpful comment. In response, we conducted additional analyses to examine the potential effects of gender using independent-samples t-tests. The results indicated that gender differences were mostly non-significant, with females scoring higher on isolation, overidentification, and competence doubt, and males scoring higher on mindfulness. These differences were also discussed in the Discussion section. The analysis of age effects was not performed, as the age distribution in our sample was narrow and homogeneous, which would not allow for meaningful comparisons.*

(7) Change "SEM" to "path analysis" since the figure of the model indicates that only manifest variables were considered.

***Authors’ response:***

*We have changed the description of the methodological basis of our model from SEM to path analysis to ensure consistency with the nature of the variables represented in the model.*

(8) Clarify the estimator used in the path analysis. MLR is recommended.

***Authors’ response:***

*We thank the reviewer for this comment. The path analysis was conducted in IBM SPSS Amos using the Maximum Likelihood (ML) estimator, which is the default estimation method in this software. As indicated in Table 4, only minor deviations from normality were observed. Given the large sample size and approximately normal distribution of the data, the use of ML estimation was considered appropriate.*

(9) Please provide syntaxes for the analyses. Currently, only a data file is provided.

***Authors’ response:***

*In response to the reviewer’s comment, we have attached all relevant syntax files used for the analyses conducted in SPSS and Amos. These files are now provided in the Mendeley Data Repository to ensure full transparency and replicability of our analytical procedures.*
