# Supplementary material for: Self-compassion and the impostor phenomenon: Associations and implications
Source: PLoS One. 2026 May 27;21(5):e0348905. doi: 10.1371/journal.pone.0348905 (PMC13215504; doi:10.1371/journal.pone.0348905)
Supplement: Attachment — Submitted filename: reviews_comments3revfin.docx [file pone.0348905.s004.docx]

**Response to Reviewers**

**Reviewer #3:**

I thank the authors for again addressing the open points and comments. Two final points remain that I kindly ask the authors to address.

(1) In response to my comment on refraining from the description of "symptoms" authors note: "We agree with the reviewer that the term “symptomatology” is somewhat inappropriate
given the nature of the IP. In line with the study cited in the corresponding paragraph,
we have replaced it with the term “syndrome.”"

However, considering that a syndrome is defined as "a group of symptoms which consistently occur together" the revision from symptoms to syndrome is inappropriate. Please just rename it "Impostor Phenomenon" in line with the standard literature and the remainder of this manuscript.

***Authors’ response:***

*We thank the reviewer for this comment. While the term “syndrome” does occasionally appear in the literature on the Impostor Phenomenon, we agree with the reviewer that its use is not appropriate in this context, because it implies the presence of a consistent grouping of symptoms, which does not align with the non-clinical nature of the construct. In line with the reviewer’s recommendation and the prevailing terminology in the field, we have therefore replaced the term with “Impostor Phenomenon” throughout the manuscript.*

(2) Since you report gender, please change "males" to "men" and "females" to "women" in line with APA recommendations on reporting gender.

***Authors’ response:***

*In accordance with APA recommendations on reporting gender, we have replaced the terms “males” and “females” with “men” and “women” throughout the manuscript. We appreciate the reviewer’s guidance in helping us improve the clarity and inclusivity of our reporting.*

**Reviewer #4:** This is my first time reviewing this manuscript, so I should begin by noting that the comments from the previous reviewer were only partially addressed. The authors clarified the use of the IPP-30, adjusted the language related to the impostor syndrome, removed the asterisks and p-values appearing in the same table, interpreted normality using skewness and kurtosis parameters, replaced the term SEM analysis with path analysis, and clarified the estimator used, although they did not apply MLR. They also made the syntax available.

1/ However, the tables could be merged and presented in a clearer, more unified format.

***Authors’ response:***

*Based on the recommendation, we carefully revised the presentation of all tables to ensure clarity, consistency, and compliance with APA formatting standards.*

*To achieve a unified structure across the manuscript, tables were reformatted to share the same graphical style as Table 1, including consistent typography and alignment. In addition, abbreviations of both questionnaires were removed from Table 1 to ensure better consistency with the remaining tables.*

*Several tables were reorganized to improve readability and conceptual coherence.
The table 2 reporting gender differences in descriptive statistics was redesigned so that gender categories appear in columns rather than rows, with separate columns for N, Mean, and SD for men and women. This format presents the information more compactly and reduces unnecessary vertical space.*

*The independent samples test table 3 was substantially simplified. In line with APA recommendations, duplicated rows generated by SPSS (“equal variances assumed / not assumed”) were removed. For each variable, we now report only the appropriate t-test, selected based on Levene’s test. A clarifying note was added below the table:*

*“Note. For each variable, Levene’s test was used to determine the appropriate t-test; only the relevant row is reported.”*

*Furthermore, to ensure full clarity and consistency across all statistical outputs, the tables 3 and were reordered to follow a uniform sequence of variables, and descriptive statistics in the table 1 were transposed so that individual variables appear in rows rather than columns. This adjustment makes the tables easier to read, aligns them with the format used elsewhere in the manuscript, and enhances comparability across sections.*

*We believe that these improvements significantly enhance the readability, coherence, and overall quality of the manuscript’s results section.*

2/ In addition, the analyses based on age and gender were only partially completed; these variables should be included as control variables in the path analysis.

***Authors’ response:***

*In response, we revised our analytical strategy as follows:*

*Gender was included as a covariate in the path analysis to evaluate the stability of the associations among latent constructs across demographic subgroups. The revised model demonstrated that the effects of gender were minimal, with standardized regression coefficients ranging from β =* –.04 to .13*. These values do not reach practical significance and did not meaningfully alter the structure or strength of the relations in the model.*

*The inclusion of gender did not change any substantive conclusions. The model remained stable, and all associations among the constructs persisted with virtually identical effect sizes. This confirms the robustness of the findings across gender influences.*

*Age was not included in the final model, and we would like to briefly clarify this decision.
Our sample exhibits extremely low age variability* (*M* = 23.46, *SD* = 3.05)*. The participants were university students within a very narrow age range.
We believe that including age as a predictor in our path analysis does not improve model validity.*

3/ I also have doubts about the use of paired t-tests to compare the positive and negative SC subscales. Since this is a cross-sectional study, I do not find a valid reason for using this type of analysis.

***Authors’ response:***

*We agree with the reviewer’s viewpoint that, given the cross-sectional nature of our study, the use of paired t-tests to compare the positive and negative self-compassion subscales is not methodologically well-justified. Since this analysis is not essential for addressing our research questions, we have removed it from the manuscript.*

*We appreciate the reviewer’s careful consideration, which has strengthened the methodological rigour of our work.*
