# Supplementary material for: Self-compassion and the impostor phenomenon: Associations and implications
Source: PLoS One. 2026 May 27;21(5):e0348905. doi: 10.1371/journal.pone.0348905 (PMC13215504; doi:10.1371/journal.pone.0348905)
Supplement: Attachment — Submitted filename: reviews_comments4rev (1).docx [file pone.0348905.s005.docx]

**Response to Reviewers**

**We sincerely thank Reviewer 3 for the careful and constructive comments, which prompted us to thoroughly re-examine the manuscript. As a result, we identified and corrected several issues that had arisen during the editing of earlier manuscript versions. Below, we respond to the reviewer’s specific comments.**

**Reviewer #3:**

(1) I am grateful to authors that they deleted instances of symptoms and syndrome to acknowledge that the IP is not a categorical variable of clinical entity. I have just discovered the sentence "The selection of this research population aligns with studies that reflect the high incidence of IP..." on p. 4 and "Several studies have focused on the incidence of IP among medical students". Since incidence is defined as "the occurrence, rate, or frequency of a disease, crime, or other undesirable thing" this should be revised accordingly.

***Authors’ response:***

**We thank the reviewer for this comment. The term “incidence” has been replaced with the less clinically loaded term “occurrence.”**

(2) Similarly, authors note "Later research pointed out the prevalence of IP regardless of gender, ethnicity, or age." Since prevalence describes the occurrence of clinical entities such as diseases, revisions are required. As noted, the IP is neither a categorical variable nor a clinical entity.

***Authors’ response:***

**The sentence was revised in line with the reviewer’s comment. Although similar terminology has been used in previous literature (e.g., “Prevalence, Predictors, and Treatment of Impostor Syndrome: a Systematic Review“ (doi:** [**10.1007/s11606-019-05364-1**](https://mcas-proxyweb.mcas.ms/certificate-checker?login=false&originalUrl=https%3A%2F%2Fdoi.org.mcas.ms%2F10.1007%2Fs11606-019-05364-1%3FMcasTsid%3D20893&McasCSRF=fe9253c5614238844ac83ef6beea786ede34dcfa23b956936b289d6a6519936b)**); „Global prevalence of imposter syndrome in health service providers: a systematic review and meta-analysis“ (doi:** [**10.1186/s40359-025-02898-4**](https://mcas-proxyweb.mcas.ms/certificate-checker?login=false&originalUrl=https%3A%2F%2Fdoi.org.mcas.ms%2F10.1186%2Fs40359-025-02898-4%3FMcasTsid%3D20893&McasCSRF=fe9253c5614238844ac83ef6beea786ede34dcfa23b956936b289d6a6519936b)**), we agree that a non-clinical formulation is more appropriate in the present context.**

(3) Tables 2 and 3: The columns N(M) and N(W) provide redundant information and can be deleted.

***Authors’ response:***

**Tables 2 and 3 have been revised in accordance with the reviewer’s comments. We used standardised statistical output, but we agree that the columns N(M) and N(W) were redundant and have therefore been removed.**

4/ Table 4: The row df is redundant.

***Authors’ response:***

**Column df was removed from Table 4.**

5/ Table 5: Half of the coefficients are redundant with the other remaining half.

***Authors’ response:***

**Table 5 was originally presented in a symmetrical format, which is standard for correlation matrices and was chosen for clarity. However, the table has been revised in response to the reviewer’s comment.**

6/ Lower and upper bounds of the confidence interval regarding SK (32.89, 54.35) do neither correspond to the b or beta coefficient (-0.28, -.07). The same is true for OI, M and other variables. This again decreases trust in the findings.

***Authors’ response:***

**Thank you for noting this inconsistency. This was a formatting error that occurred during the editing of the table. Specifically, the confidence interval values were inadvertently shifted by one row due to the inclusion of the constant row from the SPSS output. The underlying statistical results were correct, but the values were misaligned in the table. The table has now been corrected, and all confidence intervals correspond appropriately to the reported regression coefficients.**

7/ Table 7 is redundant with the coefficients given in-text. The table can be deleted.

***Authors’ response:***

**We consider Table 7 a regular summary of model fitting; however, we have removed it in response to the reviewer’s comment.**

8/ The current version of the manuscript contains 108 (!) references, which is excessive given that the study is a simple cross-sectional study investigating the associations between two variables.

***Authors’ response:***

**We provided the literature review and discussion we considered appropriate to the manuscript. Some references were also changed during the review process on the request of reviewers – the first version submitted in PLOS ONE had 78 references. The number of references reflects the need to adequately cover two theoretically and empirically distinct constructs—the impostor phenomenon and self-compassion—both of which have extensive and partly independent bodies of literature. Nevertheless, we are willing to reduce the reference list if necessary.**

9/ Reviewer also states “No” to the “5. Is the manuscript presented in an intelligible fashion and written in standard English?“.

**No specific concerns regarding language clarity or correctness were provided. We submitted a language editing certificate, and the text has not been changed in a manner that would justify a different evaluation between review rounds.**

**In the second round of the revisions (6. 10. 2025), the same reviewer responded to the same question positively:**

**“5. Is the manuscript presented in an intelligible fashion and written in standard English?

PLOS ONE does not copyedit accepted manuscripts, so the language in submitted articles must be clear, correct, and unambiguous. Any typographical or grammatical errors should be corrected at revision, so please note any specific errors here.**

**Reviewer #3: Yes“**
