# Supplementary material for: Self-compassion and the impostor phenomenon: Associations and implications
Source: PLoS One. 2026 May 27;21(5):e0348905. doi: 10.1371/journal.pone.0348905 (PMC13215504; doi:10.1371/journal.pone.0348905)
Supplement: Attachment — Submitted filename: response_reviewers_rev5_FIN.docx [file pone.0348905.s006.docx]

**Response to reviewers**

**We sincerely thank the reviewers for the careful reading of our manuscript and for the valuable and constructive comments, which helped us improve and clarify the manuscript in several respects.**

**Reviewer #5**: Conceptual contribution

**1. What the IPP30 adds conceptually (beyond psychometrics)**

***Authors’ response:***

*We addressed this issue in the Research background by adding the conceptual explanation: “The IPP30 conceptualises impostorism as a multidimensional profile with distinct facets, which aligns more closely with Clance’s original description of a constellation of impostor-related tendencies. This facet-level view allows domain-specific theorising and practical targeting (e.g., identifying which components of impostorism are most relevant for intervention), and it enables profile or typology approaches that cannot be derived from a single total score.”*

***Authors’ response:***

**2. Why competence doubt is the critical link to isolation and overidentification**

*The critical link was clarified in the Discussion – Summary of results section. “Competence doubt captures the core self-evaluative uncertainty and fear of failure that is most proximal to self-compassion processes. Isolation and overidentification reflect two mechanisms that plausibly intensify these doubts: social disconnection (I am the only one struggling) and cognitive fusion with negative self-evaluations (ruminative overinvolvement). Consistent with this rationale, competence doubt showed the strongest and most consistent associations with isolation and overidentification in our data, motivating its central role in the regression and path models.”*

***Authors’ response:***

**3. How this refines existing models**

*This comment helped us to emphasise the specificity of our results in the Discussion – Summary of results section: “Generally, our facet-level results suggest that the link between self-compassion and impostorism is not uniform but concentrated in competence doubts and the negative self-compassion dimensions. This pattern refines buffer accounts by highlighting isolation and overidentification as potential processes associated with competence-related impostor doubts.”*

**4. Path model: implies mechanism and directionality.**
Although the authors appropriately note that directional paths are not causal, the interpretation of indirect pathways and the narrative sequencing of variables implies a process model that cannot be supported with cross-sectional data.
Several paths are plausibly reversible or reciprocal, particularly the links between isolation and overidentification, competence doubt and working style, and competence doubt and other-self divergence. The cited literature supports these variables’ covariance but does not establish temporal ordering and the unidirectional paths are not sufficiently justified vs other plausible directionality that are not tested.
I recommend testing and reporting fit comparisons for at least one plausible alternative directional model for each key pathway, and/or providing a stronger justification for why the chosen ordering is privileged over alternatives
As currently presented, the directional arrows risk implying mechanistic interpretation that cannot be supported by the present design.

***Authors’ response:***

*We thank the reviewer for the suggestion to test alternative directional specifications of the relationship between overidentification and isolation. In response, we estimated an alternative model (Model_alt1) in which the direction of this path was reversed. This specification showed a substantially poorer model fit and did not adequately represent the data. Importantly, engaging with this alternative prompted us to reconsider the hypothesised status of this association. Given the reciprocal nature suggested in the literature, we revised the model to represent the overidentification–isolation link as a covariance rather than a directional path. This specification proved both theoretically more appropriate and empirically adequate, yielding a slightly improved model fit (Model_current). Similarly, we tested the association between Common Humanity and Self-Kindness as a covariance. However, this specification was not supported empirically, as some of the correlation and regression coefficients were low and did not provide sufficient empirical support for this representation of the relationship (Model_alt4).*

*In addition, we tested alternative directional specifications for the remaining key pathways (Models_alt2 and alt3). However, these models likewise showed poorer fit and did not improve the overall representation of the data.*

| **Fit Index** | **χ²/df** | **CFI** | **TLI** | **RMSEA** | **AIC (Our model)** | **And AIC (Saturated model)** |
| --- | --- | --- | --- | --- | --- | --- |
| Model_previous | 2.02 | .984 | .974 | .041 | 70.25 | 70.000 |
| Model_alt1 | 18.67 | .72 | .55 | .172 | 286.65 | 70.000 |
| Model_current | 1.57 | .99 | .99 | 0.31 | 64.78 | 70.000 |
| Model_alt2 | 19.02 | .74 | .54 | .173 | 274.2 | 70.000 |
| Model_alt3 | 21.25 | .71 | .48 | .184 | 300.97 | 70.000 |

**Model_alt1**

**Model_current**

**Model_alt2**

**Model_alt3**

**Model_alt4**

**5. The choice to only regress competence doubt feels post hoc- justify why the choice was made not to regress other facets of IP. Not including the rest of the IP subscales undermines the use of the IPP30. This is a key to what this study adds to the literature.**

***Authors’ response:***

*We thank the reviewer for this important and thoughtful comment. We agree that focusing exclusively on competence doubt without examining the remaining IP facets could raise concerns regarding the full use of the IPP30. In response, we extended the regression analyses to include the other IP subscales and now report the relevant results in the Results and Discussion sections. This additional analysis demonstrates that competence doubt occupies a particularly central role within the network of relationships with self-compassion dimensions, while the remaining IP facets show more limited – but in some cases significant - associations.*

**6. Gender was analyzed and reported but not well interpreted. This factor was also not included in hypotheses. It felt like an important element in the results but then dropped. I suggest either reducing the emphasis in the results or discussing the differences in more detail and potentially adding a hypothesis.**

***Authors’ response:***

*Upon further consideration, we agree that the inclusion of gender was not sufficiently integrated into the theoretical framework, as it was not specified a priori in the hypotheses. To maintain theoretical coherence and avoid overemphasising exploratory findings, we therefore decided to remove the gender-related SEM analysis from the main model and results. However, we kept the analysis of the gender differences in IP and SC for a deeper descriptive frame of our research sample, commenting on them and further research direction of this field in the Discussion.*

**7. Over cited paper- reduce the number of citations if they are redundant.**

***Authors’ response:***

*Following a careful review of the reference list, redundant citations were removed, reducing the total number of references from 108 to 95.*

**8. Review paper- particularly discussion for redundancies. Some content is repeated with only minor rephrasing.**

***Authors’ response:***

*We carefully reviewed the Discussion section for redundancies and reduced repeated content across sections, particularly where similar interpretations of the negative self-compassion subscales and impostor phenomenon were presented with minor rephrasing. The revised Discussion now provides a more concise and clearly structured interpretation of the findings.*

**9. Flow of introduction/background could improve. Connecting sentences would help connect constructs.**

***Authors’ response:***

*We restructured part of the section and added/reformulated connecting sentences to support the flow of the text.*

**Reviewer #6:** This manuscript addresses a relevant topic by linking evolutionary perspectives with current mental health constructs. While the subject is of interest, the conceptual integration between these frameworks would benefit from greater clarity.

**1. In particular, the distinction between the impostor phenomenon and self-compassion should be more explicitly defined and theoretically grounded.**

***Authors’ response:***

*We thank reviewer 6 for their valuable comments, which improved the clarity of our text and the reproducibility of our findings. In response to the first comment, we revised the Introduction to more explicitly and theoretically delineate the distinction between the impostor phenomenon and self-compassion. Specifically, we clarify that the impostor phenomenon reflects a predominantly negative and performance-contingent self-evaluation characterised by relatively specific cognitive–emotional patterns, whereas self-compassion represents a broader, multifaceted self-attitude that shapes how individuals respond to experiences of failure and inadequacy. (…)*

2**. The methodology describes analytical parameters, inclusion criteria, and scale validation procedures adapted to the target population. However, the manuscript would benefit from additional detail regarding the sampling strategy, participant characteristics, and the rationale for selecting the measurement instruments, in order to improve transparency and reproducibility.**

***Authors’ response:***

*We added details regarding the sampling strategy and participant characteristics* *(section Procedure). Further participant characteristics were not assessed to ensure anonymity and encourage voluntary participation. We also clarified the rationale for instruments by emphasizing their psychometric quality, availability in validated Czech versions, and their conceptual alignment with the theoretical framework and research design of the study (section Measures).*

**3. With respect to the presentation of the results, the formatting of the tables should be improved; specifically, centering the tables would enhance readability and consistency.**

***Authors’ response:***

*We centred all tables to improve readability and ensure formatting consistency throughout the manuscript.*

**4. Although the results, analysis, and discussion are generally aligned with the reported numerical findings, the interpretation remains largely descriptive. A more critical engagement with the data, including a clearer discussion of variability, limitations, and potential sources of bias, would strengthen the manuscript.**

*We thank the reviewer for this helpful comment and for highlighting the need for a more critical engagement with the data. In response, we expanded the interpretation of the results in several parts of the manuscript to provide a more nuanced discussion of the observed patterns.*

*First, we elaborated on the interpretation of the descriptive statistics. In addition to reporting means and standard deviations, we now discuss the variability of the variables more explicitly and interpret the skewness and kurtosis values.*

*Second, we expanded the interpretation of the correlation coefficients, discussing their relative magnitude and implications for understanding the specific facets of self-compassion associated with competence-related impostor doubts.*

*Third, we elaborated on the interpretation of the regression results. In addition to reporting the regression coefficients and their statistical significance, the revised text now also discusses the corresponding confidence intervals and their implications for the precision and stability of the estimated effects.*

*Finally, we expanded the discussion of study limitations and potential sources of bias. In addition to the previously discussed limitations related to the cross-sectional design, which does not allow causal inferences, and the characteristics of the sample (participants from a single faculty, predominantly students, with limited national diversity and gender imbalance), we added a more explicit discussion of potential methodological biases related to the sampling strategy and the use of self-report and self-reflective measures. In this context, we also address the possible influence of common method variance, which may inflate the observed associations.*

*We believe that these revisions provide a more critical interpretation of the findings and strengthen the overall discussion of the results.*
