# Supplementary material for: Self-compassion and the impostor phenomenon: Associations and implications
Source: PLoS One. 2026 May 27;21(5):e0348905. doi: 10.1371/journal.pone.0348905 (PMC13215504; doi:10.1371/journal.pone.0348905)
Supplement: Attachment — Submitted filename: response_reviewers_rev6_FIN.docx [file pone.0348905.s007.docx]

**Response to reviewers**

We sincerely thank the reviewers for their supportive comments and suggestions, which have helped us to improve the methodological rigour and clarity of the manuscript.

**Reviewer #5**:

**1. Table 1 could remove skew kurtosis as they are not a problem in this data. You mentioned the range in the narrative and that is sufficient.**

***Authors’ response:***

*Thank you for this constructive suggestion. The skewness and kurtosis columns have been removed from Table 1. Please note that this change could not be displayed in tracked changes mode because it involved deleting table columns.*

**2. Combine table 2 and 3 would make it easier to follow the M scores and significance of t-tests.**

***Authors’ response:***

*We agree that combining Tables 2 and 3 improves the clarity and readability of the results in this section, and we have merged the tables accordingly.*

**3. I don’t feel table 4 is necessary- just reporting that while many variables significantly departed from normality (common with large samples) the skew/kurtosis values were within acceptable range.**

***Authors’ response:***

*We agree with this comment, and Table 4 has been removed. The relevant interpretation has been incorporated into the text.*

**4. Report VIF to resolve multicollinearity concerns in the regression. I wondered why the correlations between self-kindness mindfulness and competence doubt were significant but that was lost in regression. This could be shared variance vs a true non-significant finding. Just nuance if you want to add that angel and discuss further.**

***Authors’ response:***

*Thank you for this observation. We examined multicollinearity and found that VIF values ranged from 1.41 to 1.76, indicating no concerns. The discrepancy between the significant correlations and non-significant regression coefficients likely reflects shared variance among predictors rather than multicollinearity.*

**5. One last review to make sure causal language is tempered.**

***Authors’ response:***

*Thank you. Although we had already reduced the use of causal language in previous revisions, we conducted an additional careful review of the manuscript and further revised several phrases that could imply causality.*
